# Mechanical properties and chemical synergistic mechanism of lithium slag concrete under mechanical-chemical activation conditions

**DOI:** 10.1038/s41598-026-48058-5

**Published:** 2026-04-14

**Authors:** Yongjie Yan, Daoxue Yang, Xuanzi Wang, Zhi Liu, Lei Feng, Shiyu Wei, Xiankang Luo, Tong Xiao

**Affiliations:** 1 Jiangxi Academy of Water Science and Engineering, Nanchang, 330029 Jiangxi China; 2 School of Civil and Surveying & Mapping Engineering, Jiangxi University of Science and Technology, Ganzhou, 341000 Jiangxi China

**Keywords:** Lithium slag concrete, Alkali-mechanically activation, Compressive strength, Microstructure, Pozzolanic reaction, Chemistry, Engineering, Materials science

## Abstract

This study systematically examines the mechanical performance and microstructural evolution of concrete incorporating alkali-mechanically activated lithium slag (LS). Unconfined compressive strength (UCS) tests were conducted at 7 and 28 days of curing to assess the influence of LS content on early and later-age strength development. Multiscale characterization techniques, including scanning electron microscopy (SEM), X-ray Fluorescence (XRF), X-ray Diffraction (XRD) nuclear magnetic resonance (NMR), and Fourier-transform infrared spectroscopy (FTIR), were used to reveal the dual mechanism of chemical activation and physical pore refinement. The results show a non-monotonic trend in compressive strength with increasing LS content, with an optimum at 20% replacement, where the UCS reached 103% and 105% of that of the control mix at 7 and 28 days, respectively. NMR analyses reveal that activated LS contributes to refinement of the pore structure, resulting in reduced pore size and enhanced matrix densification. SEM, energy dispersive spectroscopy (EDS), and thermogravimetric (TG) analyses indicate that the improved strength is primarily attributed to the pozzolanic reaction between activated LS and cement hydration products, forming additional calcium silicate hydrate (C-S-H) and other binding phases. These findings highlight the dual role of alkali-mechanically activated LS in enhancing concrete performance through coupled microstructural refinement and chemical reactivity, offering a sustainable approach for valorizing lithium slag in cementitious materials.

## Introduction

 The consumption of lithium resources has increased exponentially worldwide due to the new energy vehicle industry’s rapid development^1–5^. Industrial solid waste LS, generated from the lithium resource smelting process, reaches an annual output of 12 million tons^6,7^. The efficient resource utilization of LS has become a technical bottleneck restricting the sustainable development of the lithium battery industry chain. It is critically important to develop innovative value-added solutions to ensure closed-loop management of solid waste^8,9^. Currently, an increasing number of materials are being utilized in concrete^10–13^.

Lithium extraction from lepidolite (Li₂O·Al₂O₃·4SiO₂) using sulfuric acid generates lithium sulfate (Li₂SO₄), aluminum sulfate (Al₂(SO₄)₃), amorphous silica (SiO₂), and water. The process involves acid leaching with 85% H₂SO₄, which destroys the lepidolite crystal structure, leaving behind a solid waste (LS) composed mainly of amorphous SiO₂ and inert γ-Al₂O₃^14–18^. However, LS retains significant sulfate residues, limiting its applicability in cementitious systems. The pozzolanic activity of LS, a key factor in its cementitious properties, is considerably lower than that of conventional supplementary cementitious material (SCM) such as fly ash^19,20^. Moreover, the residual sulfate in the process further limits the applicability of its auxiliary cementation system by inhibiting the hydration of C₃A^21^. Current LS valorization strategies focus on two main approaches. Partial cement replacement: LS can act as an SCM, enhancing early-stage concrete strength. However, due to its low pozzolanic reactivity, the 28d strength improvement remains limited^22–24^. Geopolymer synthesis: High-energy ball milling or alkali activation modifies LS’s physicochemical properties. These methods disrupt inert aluminosilicate networks, improving reactivity^25–27^. Additionally, LS can serve as a fine aggregate filler, enhancing concrete durability by densifying the pore structure^28,29^.

Coal gangue and fly ash decrease hydration heat, increase concrete workability, and postpone initial setting time. They usually only produce 70.8% of the control group’s 28d strength, though, due to their low pozzolanic reactivity^30,31^. Volumetric instability limits the cementitious qualities of copper and steel slag^32–34^. Although silica fume improves strength and durability, it also raises expenses and water requirements^35^. On the other hand, LS, an industrial solid waste of lithium extraction, has advantages for the environment in addition to pozzolanic reactivity. LS is a viable candidate for partial replacement in concrete since it is an industrial waste that offers a cost-effective and environmentally friendly substitute for traditional cementitious additives.

LS has a significant amount of amorphous SiO₂ (more than 25%) but less reactive CaO (less than 20%). Due to its limited pozzolanic reactivity, this composition limits its ability to increase the strength of concrete^25^. Nevertheless, LS reactivity is greatly increased by alkali activation with sodium hydroxide (NaOH), which makes it more appropriate for geopolymer production^26^. According to durability studies, concrete with 40% LS replacement has a 180d compressive strength that is 18.34% higher than that of regular concrete. Together with the creation of more pozzolanic reaction products, the improved pore structure is responsible for this improvement, as overall porosity drops by 20.2% after 180 d^28^.

Compressive strength and chemical resistance are two important geopolymer characteristics that are successfully enhanced by alkali activation technique. LS aluminosilicates’ Si-O and Al-O bonds are broken by hydroxyl attack when they are subjected to high alkalinity (pH ≥ 11.5). LS can actively participate in alkali-activated reactions thanks to its depolymerization process^22^. By greatly increasing its surface area, mechanical activation further improves LS reactivity^36–39^. This enhancement is shown in alkaline, sulfate, and composite solutions, among other conditions. In cementitious systems, the activated LS particles exhibit exceptional hydration performance. Free Ca(OH)₂ and mechanically activated LS interact via dissolution-precipitation processes during cement hydration. Calcium silicate hydrate (C-S-H) gels with optimal polymerization degrees are the result of this reaction^40–43^. The pore structure of cementitious materials is efficiently refined by the resultant gel network, improving their mechanical qualities^44–47^.

This study uses a mechano-alkali synergistic activation technique to unleash LS reactivity to develop the theoretical framework of LS-incorporated concrete under synergistic mechanical activation and alkali excitation. In contrast to NaOH activation, which produces ≡ Si–O⁻/≡Al–O⁻ active sites that facilitate C-(A)-S-H nucleation, high-energy ball milling produces defective LS surfaces. Using TG, SEM in conjunction with EDS, and X-ray fluorescence (XRF), the pozzolanic reaction processes between LS and cementitious materials were examined.

## Experimental material and scheme

### Experimental material

**Fig. 1:**
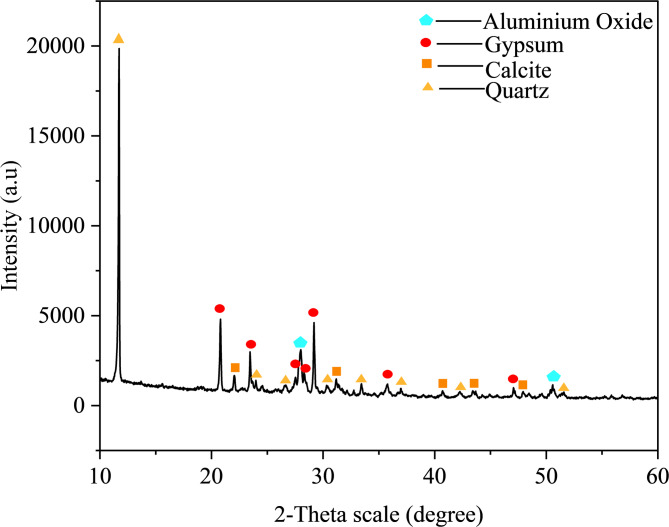
XRD pattern of LS.

This study systematically investigates the activation mechanism of lithium slag (LS) through the synergistic effects of alkali excitation and mechanical activation. LS is a solid waste byproduct generated during lepidolite ore processing in Yichun City, Jiangxi Province. A series of LS concrete specimens were prepared to evaluate the performance evolution under varying LS content conditions. The experimental materials included gravel (10–26 mm particle size), fine sand (fineness modulus of 2.56), mechanically activated LS, and ordinary Portland cement (P.O. 42.5). X-ray fluorescence (XRF) spectroscopy was employed to characterize the major elemental composition of LS, and the quantitative results are presented in Table 1. The XRD pattern of LS is shown in Fig. 1.

**Table 1:** Chemical compositions of LS (wt%).

Materials	CaO	SiO_2_	Al_2_O_3_	Fe_2_O_3_	MgO	Na_2_O	SO_3_	Other
LS	15.10	26.92	17.42	2.23	1.03	2.57	19.17	15.56
Cement	63.46	20.94	4.31	3.28	2.76	0.56	2.23	2.46

Four major components were found in LS by XRF analysis: calcium oxide (CaO, 15.10 wt%), silicon dioxide (SiO₂, 26.92 wt%), aluminum oxide (Al₂O₃, 17.42 wt%), and sulfur trioxide (SO₃, 19.17 wt%). The corresponding elemental compositions calculated from these oxide percentages are: calcium (10.8%), silicon (12.6%), aluminum (9.2%), and sulfur (7.7%). Table 1 presents the chemical composition of LS obtained from XRF spectroscopy. XRF analysis reports elemental composition in the form of oxides (e.g., CaO, SiO₂, Al₂O₃) as a standard convention for expressing chemical composition of materials. The CaO value of 15.10 wt% in Table 1 represents the total calcium content expressed as calcium oxide equivalent. XRF provides elemental quantification but does not identify the actual mineral phases or chemical speciation of elements in the sample.

**Fig. 2:**
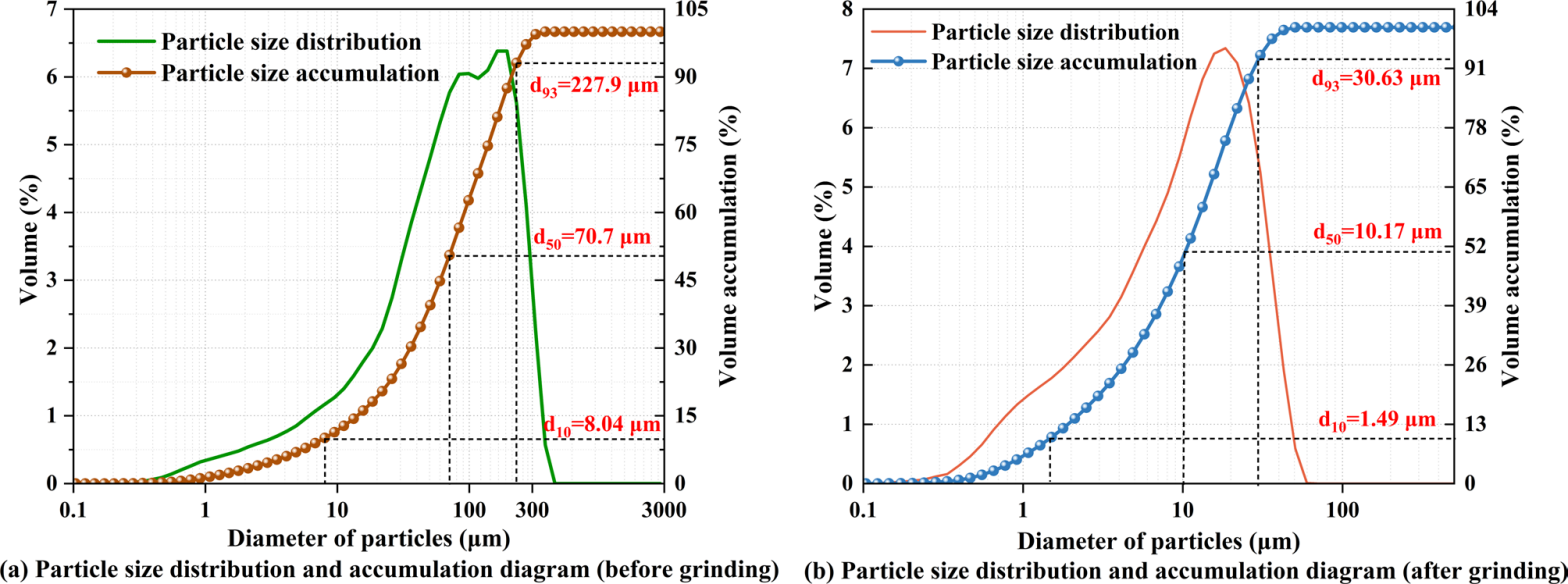
Particle size distribution and accumulation of LS.

Using laser diffraction particle size analysis, the particle size distribution properties of LS were statistically examined both before and after wet grinding treatment. The findings are shown in Fig. 2. The experimental findings showed that wet grinding significantly improved the particle system’s refinement. Significant decreases were seen in the typical diameters: *d*₁₀ dropped from 8.04 μm to 1.49 μm (81.5% reduction), *d*₅₀ dropped from 70.7 μm to 10.17 μm (85.6% reduction), and *d*₉₃ dropped from 227.9 μm to 30.63 μm (86.6% reduction).

Effective disintegration of LS agglomerates was indicated by a shift to the left in the particle size distribution curve. Particle size changed from a coarse-grained condition (> 200 μm) to a submicron-to-micron scale (1–30 μm) because of this mechanical activation. It was discovered that the obtained refinement improved the LS materials’ specific surface area and interfacial reactivity.

### Preparation of concrete specimens

Wet grinding with multi-sized ZrO₂ grinding media (mass ratio 8:54:145 for large-medium-small balls) was used in the LS refinement process in a planetary ball mill. 400 g of LS were added to four alumina jars at a 3/5 filling ratio, and the jars were then immersed in deionized water. The grinding process lasted 45 min at 400 rpm^48^. The resultant slurry underwent 12 h gravity sedimentation after first being wet-sieved through a 300-mesh screen. Following the removal of the supernatant, the material was oven-dried for 3 h at 105 °C (ASTM C110). The refined LS was obtained through a final secondary grinding phase that took 30 min.

**Fig. 3:**
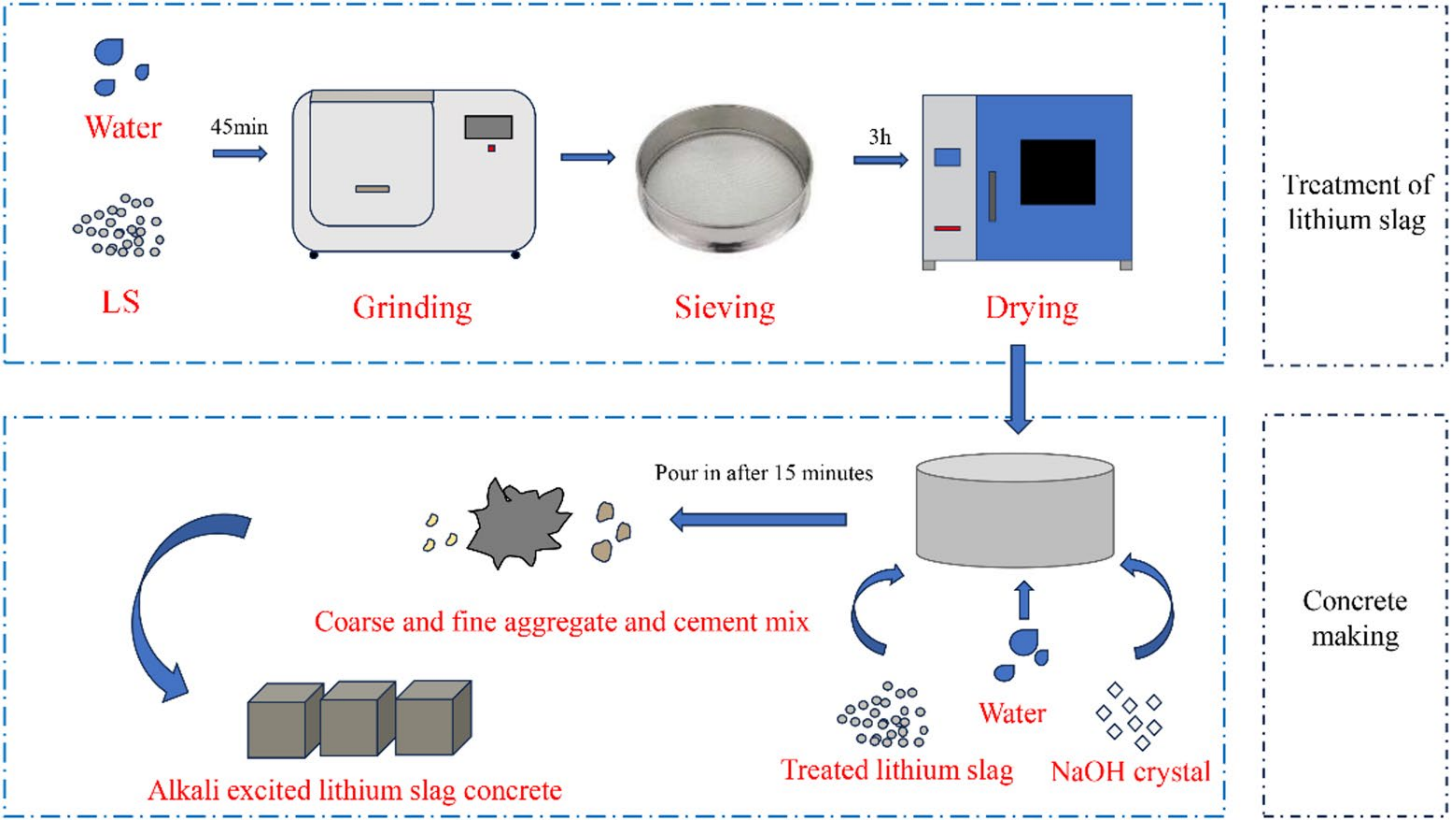
Preparation process of LS concrete specimen.

Figure 3 illustrates the preparation process of LS concrete specimens with varying LS-to-cement replacement ratios. In this study, LS and ordinary Portland cement together constituted the cementitious material, with a constant water-to-binder ratio maintained across all mixtures. The LS content was increased at the expense of cement, while the total binder mass and water dosage remained unchanged. Before mixing, the mechanically activated LS was accurately weighed, and the coarse aggregates (gravel) were oven-dried at 108 °C for four hours. To evaluate the effect of LS content on mechanical properties and microstructure, concrete specimens were prepared with LS replacement ratios of 10%, 15%, 20%, 25%, and 30% by mass of the total binder (Table 2). A control group without LS was also prepared for comparison.

An alkaline activator solution was prepared by dissolving NaOH solids in deionized water under magnetic stirring until a hydroxide ion concentration of 0.01 mol/L was achieved. Cement and coarse/fine aggregates were mechanically mixed for three minutes during the dry mixing process. Two batches of the NaOH-LS suspension were added during the wet mixing phase, which was then followed by thorough blending and vibration compaction. Three replicate specimens were produced per group to reduce data scattering effects.

**Table 2:** Mix ratio of a single LS concrete specimen.

Group ID	Substitution rate	Cement	LS	Water	Sand	Gravel
0%LS	0%	126.0 g	0.0 g	81 g	257 g	500 g
10%LS	10%	113.4 g	12.6 g	81 g	257 g	500 g
15%LS	15%	107.1 g	18.9 g	81 g	257 g	500 g
20%LS	20%	100.8 g	25.2 g	81 g	257 g	500 g
25%LS	25%	94.5 g	31.5 g	81 g	257 g	500 g
30%LS	30%	88.2 g	37.8 g	81 g	257 g	500 g

### Test methods

#### Mechanical test

**Fig. 4:**
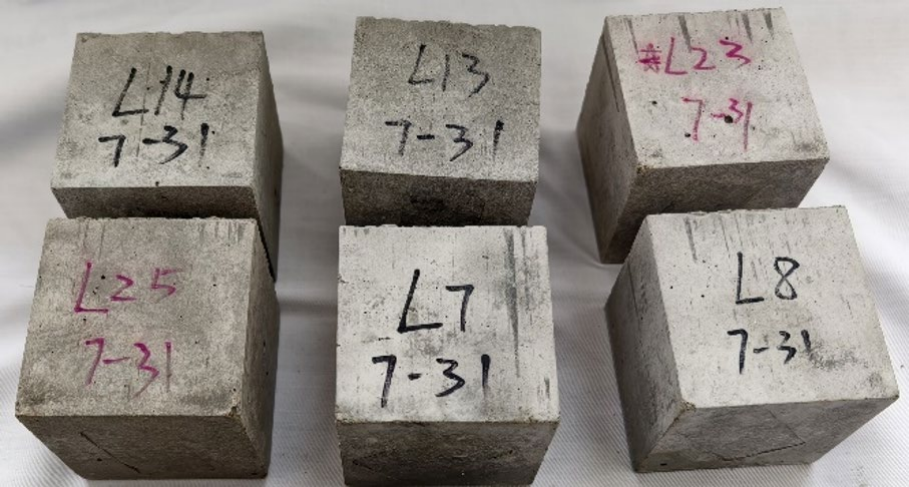
Part of the appearance of LS concrete.

The compressive strength of concrete specimens with varying LS contents was tested in accordance with GB/T 17,671 − 2021. Cubic specimens measuring 70.7 mm × 70.7 mm × 70.7 mm (Fig. 4) were cured under standard conditions (temperature of 20 ± 1 °C and relative humidity of not less than 95%) for 7 and 28 days. The strength tests were conducted using a WDW-300 electronic universal testing machine at a constant loading rate of 0.5 mm/min. For each LS replacement ratio and curing age, three replicate specimens were prepared to ensure statistical reliability. The reported results represent the arithmetic mean of valid measurements, with individual data points deviating by more than 15% from the mean value being excluded.

By comparing the compressive strengths of the control and LS-incorporated groups, Eq. 1 was used to determine the strength activity index (SAI) of LS concrete:

1 $$SAI=\frac{{{\sigma _c}}}{{{\sigma _0}}}$$

Where, ${\sigma _c}$ represents the uniaxial compressive strength of LS concrete specimens, and ${\sigma _0}$ denotes the corresponding value of control specimens.

#### Low-field nuclear magnetic resonance

Concrete specimens were subjected to non-destructive pore structure characterization using a MesoMR23-060 H low-field NMR analyzer (Niumag Electronic Technology, China). Concrete specimens with LS inclusion rates of 0%, 10%, 15%, 20%, 25%, and 30% were subjected to vacuum saturation treatment. By submerging saturated specimens in absolute ethanol (purity ≥ 99.8%), hydration termination was accomplished.

Prior to testing, the magnet was thermally stabilized at 35 ± 0.5 °C, with magnetic field homogeneity calibrated using standard reference specimens. Specimens washed with ethanol were placed in the center of radiofrequency coil. Data acquisition using the Carr-Purcell-Meiboom-Gill (CPMG) pulse sequence with defined parameters: echo time = 0.2 ms, echo number = 4096, repetition interval = 3000 ms.

The Simultaneous Iterative Reconstruction Technique (SIRT) technique was used to rebuild T₂ relaxation spectra. Features of the pore size distribution and porosity were statistically examined at various amounts of LS incorporation. To prevent anisotropy effects, measurements were made in triplicate for each group, maintaining a data acquisition accuracy of ± 0.5%.

#### Fourier transform infrared spectroscopy (FTIR)

A Bruker Alpha FTIR spectrometer (Germany) with a resolution of 4 cm⁻¹ was used to study the evolution of chemical bonds in hydration products. Concrete specimens with varying ratios of LS inclusion were used to extract cement mortar matrices, which were then cryo-fractured in liquid nitrogen and crushed to particle sizes less than 2 μm (as confirmed by a Malvern particle size analyzer).

200 mg of powder and pre-dried KBr (spectroscopic grade, 150 °C for 2 h) were combined in an agate mortar at a 1:100 mass ratio for 30 min of dry mixing to prepare the sample. Transparent pellets (φ13 mm) with a thickness of 0.2 ± 0.02 mm and a transmittance of more than 70% were created by pressing the mixture under 10 MPa.

The spectral acquisition parameters were set up as follows: a DTGS detector received the signals, 32 cumulative scans, and a wavenumber range of 400–4000 cm⁻¹. Savitzky-Golay smoothing (9-point window) and automatic baseline correction were used to treat the raw spectra. Peak assignments were carried out using the OPUS software’s HRAM Atlas database, with particular attention paid to quantitative study of changes in intensity of the following distinctive bands: asymmetric stretching vibrations of Si-O-T (T = Si/Al) (950–1200 cm⁻¹), bending vibrations of Ca-OH (3640 cm⁻¹), and antisymmetric stretching vibrations of CO₃²^−^ (1420–1480 cm⁻¹). To reduce preparation-induced variations, each sample was prepared in triplicate. Throughout the measurements, the wavenumber calibration accuracy was kept within ± 0.5 cm⁻¹.

#### Scanning electron microscope (SEM)

Fresh fracture surfaces were generated from cement matrix regions free of aggregate interference using liquid nitrogen cryo-fracturing. To provide structural support, the non-observation surfaces were encased with low-viscosity epoxy resin (< 0.2% curing shrinkage) and vacuum-cured at 60 °C for 12 h. The fracture surfaces were polished using 5 keV Ar⁺ ion beams at a 5° incidence angle before microscopic examination. To avoid charging effects during characterization, an ultra-high vacuum sputter coating was used to form a 5 nm Au conductive layer.

Microstructural characteristics of 28-day-cured samples with varying lithium slag incorporation levels were examined using backscattered electron (BSE) imaging on a FEI Nova Nano SEM 450 field emission scanning electron microscope. For the best imaging circumstances, the device was tuned at 1.6 nA probe current and 15 kV acceleration voltage. With an Octane Elite SDD detector, elemental mapping was carried out while keeping a 60 s live time and count rates higher than 20 kcps. SmartMAP mode was used to acquire high-resolution images (1024 × 884 pixels) with a pixel dwell duration of 30 µs. Selected micro-regions were subjected to phase composition analysis using the INCA program from Oxford Instruments.

#### Thermal analysis (TG-DTG)

Thermogravimetric analysis was performed using a Netzsch STA 449 F3 instrument. The hydration products were ground in absolute ethanol to achieve *d*₉₀≤5 μm, as confirmed by laser particle size analysis. The ground samples were then vacuum-dried at 105 °C for 24 h to remove physically adsorbed water. Samples weighing 8.50 ± 0.05 mg were carefully loaded into PtRh crucibles, and the temperature program consisted of heating from 50 °C to 900 °C at a constant rate of 10 K/min under flowing nitrogen (50 mL/min).

Proteus software was used for data processing, which included thermal hysteresis correction and baseline correction (empty crucible compensation method). Derivative thermogravimetric (DTG) curves were subjected to Gaussian-Lorentzian deconvolution to detect distinctive phase transition regions: Ca(OH)₂ dehydroxylation (400–500 °C) and CaCO₃ breakdown (600–800 °C). For every sample group, three measurements were made, and the precision of the mass measurement was kept within ± 0.1 µg.

## Results and analysis

### Compressive strength

**Fig. 5:**
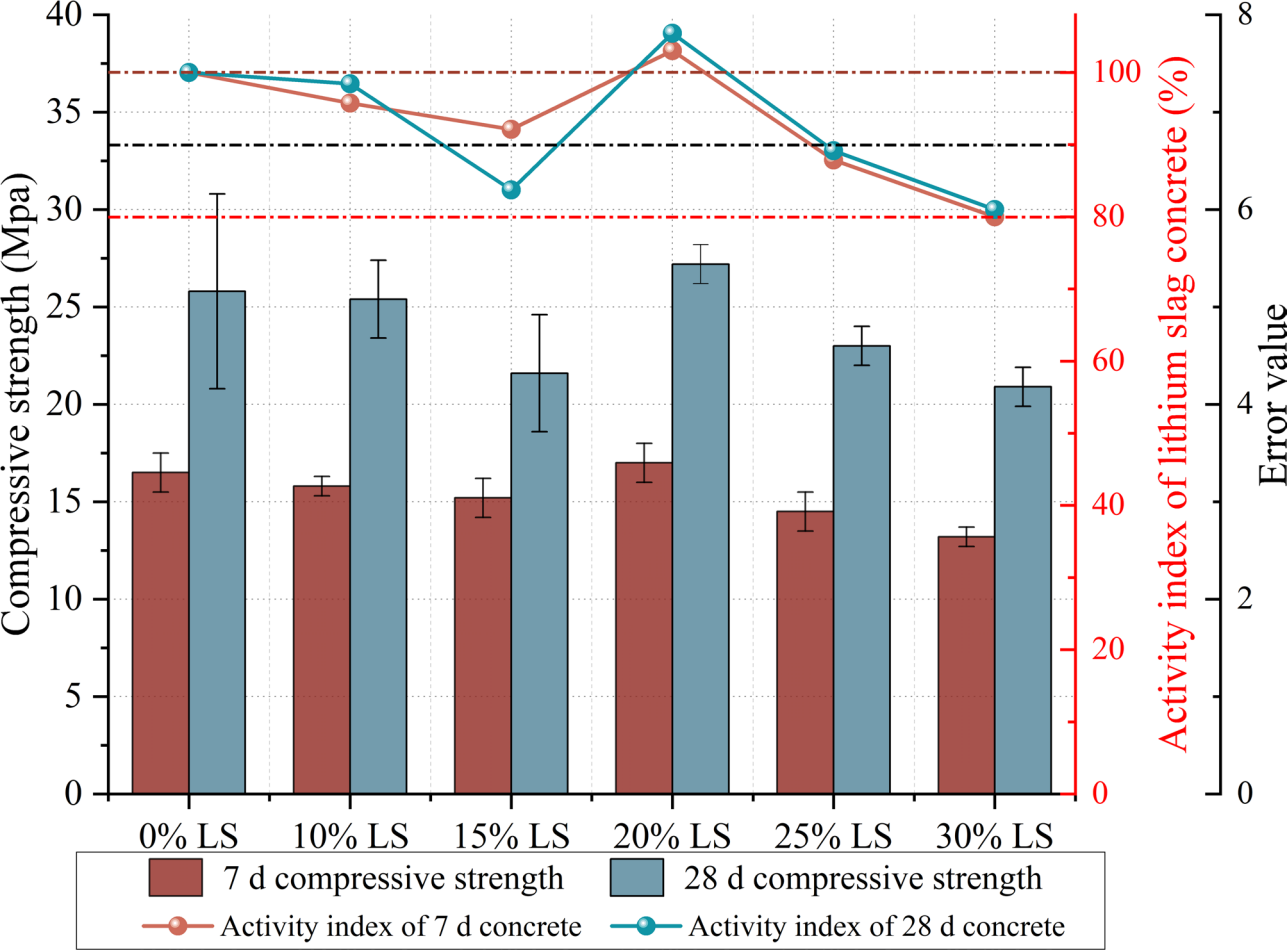
Compressive strength and activity index of LS concrete.

This study looked at the effects of LS content on the mechanical characteristics of concrete at various curing ages. Specimens containing 0%, 10%, 15%, 20%, 25%, and 30% LS underwent uniaxial compression tests. The findings, which are displayed in Fig. 5, show distinct patterns in the growth of strength.

The mechanical properties of concrete specimens with the proper levels of LS replacement were found to be improved by the combined effects of mechanical activation and alkali excitation, as shown in Fig. 5. The 20% LS replacement group’s compressive strengths increased to 17 MPa (7 d) and 27.2 MPa (28 d), while the control group’s compressive strengths were 16.5 MPa and 25.8 MPa. This resulted in strength gain rates of 3.0% and 5.4%, respectively. With reference to the control group (0% LS, compressive strength of 16.5 MPa), the compressive strength exhibited a gradual decreasing trend as the LS content increased up to 20%. At 7 days of curing, the strengths were 15.8 MPa and 15.2 MPa for LS replacement ratios of 10% and 15%, respectively; at 28 days, the corresponding strengths were 25.4 MPa and 21.6 MPa. Beyond 20% replacement, a more pronounced decline in strength was observed. At 7 days, the strength further decreased to 14.5 MPa (25% LS) and 13.2 MPa (30% LS), while at 28 days, the values dropped to 23.0 MPa and 20.9 MPa, respectively.

According to the experimental findings, the combined effects of alkali excitation and mechanical activation were unable to make up for the strength loss brought on by cement depletion at replacement levels of 10% and 15%. Lower compressive strengths were observed in comparison to control specimens because the pozzolanic reaction degree and filling effect of LS at these conditions were insufficient to counteract the decreased cement content^2^. The improved pozzolanic reactions in alkaline conditions produced ideal compressive strengths of 17 MPa (7 d) and 27.2 MPa (28 d) at the 20% replacement level. However, as the LS concentration increased, strength gradually declined when replacement above 20%. This phenomenon is caused by the high sulfate (SO₄²⁻) concentration in LS as well as the restricted pozzolanic reactivity and filling effect. The compressive strength of LS-incorporated concrete was weakened by expansive phases like ettringite (AFt), which were produced by chemical interactions between hydration products and sulfate ions. The volumetric expansion of these phases deteriorated the pore structure inside concrete specimens^49,50^.

The high SO₃ content (19.17%) in LS significantly exceeds the typical limits specified in cement standards. According to ASTM C150/C150M-23, the maximum SO₃ content for Type I Portland cement is 3.0% for cement with C₃A ≤ 8%, and 4.5% for cement with C₃A > 8%. When LS is incorporated at 20% replacement level, the equivalent SO₃ contribution to the cementitious system is approximately 3.83% (19.17% × 20%), which approaches the upper limit of ASTM C150 specifications.

To mitigate potential sulfate-related issues, the alkali activation process (pH = 12) in this study promotes early formation of ettringite (AFt) during the initial hydration stage rather than delayed formation. This controlled early formation strategy prevents the detrimental effects of delayed ettringite formation (DEF) that typically occurs when sulfate reacts with hydrated calcium aluminate phases at later ages.

Furthermore, the mechanical activation (wet grinding) reduces the particle size of LS to *d*₅₀ = 10.17 μm, which enhances the dissolution kinetics of sulfate ions. This allows sulfate to participate in early hydration reactions before the cement paste develops significant strength, thereby minimizing internal expansion stresses. The optimal replacement level of 20% was determined based on the balance between pozzolanic reactivity enhancement and sulfate-induced expansion control.

### NMR

**Fig. 6:**
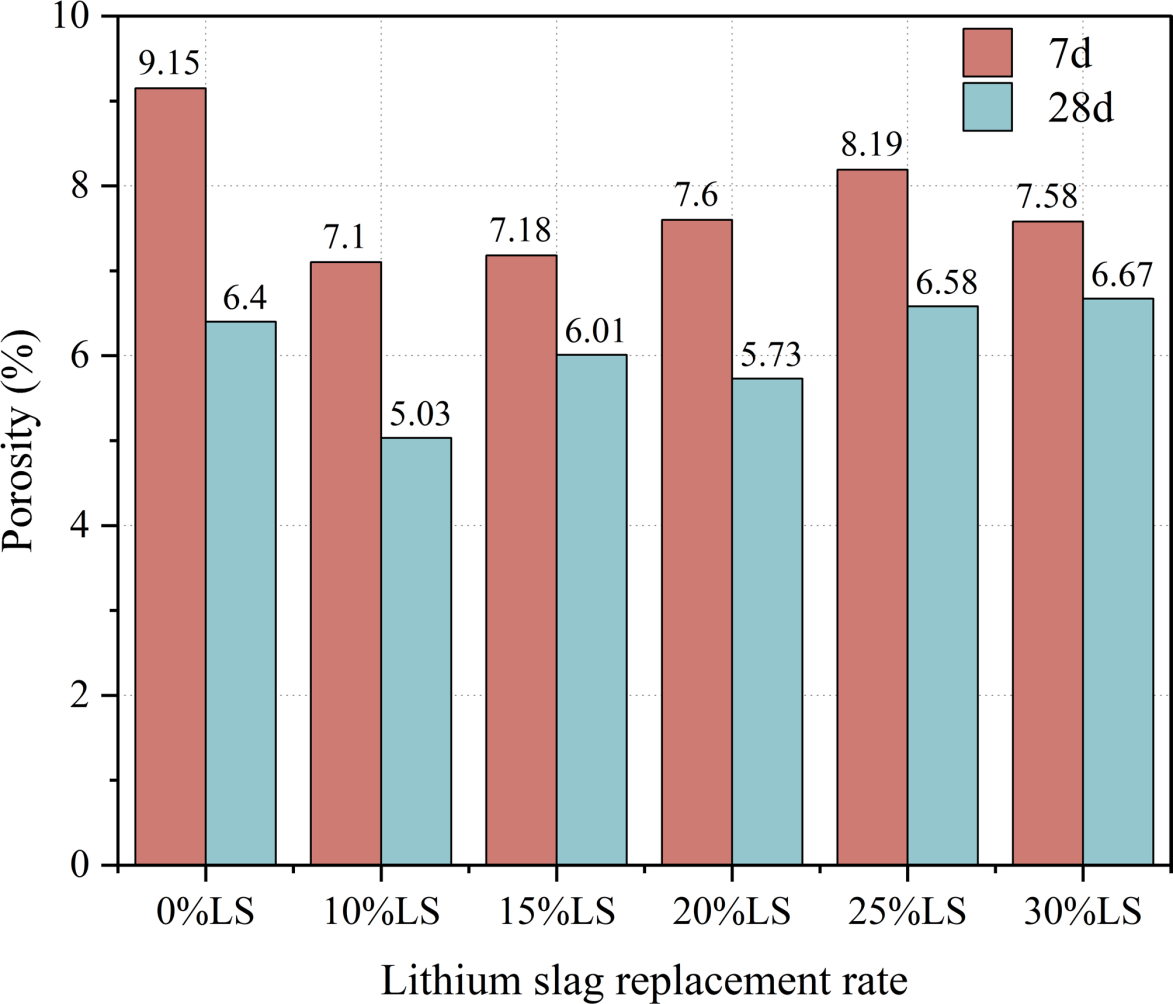
Porosity of LS concrete with different dosage.

NMR spectroscopy was used to statistically quantify the porosity of concrete specimens to examine the impact of LS incorporation on the evolution of pore structure under various curing ages. Standard conditions were used to prepare and cure specimens with LS replacement levels of 0%, 10%, 15%, 20%, 25%, and 30% for 7 and 28 d. Figure 6 displays the findings of the quantitative porosity analysis.

As shown in Fig. 6, a notable nonlinear interdependence between LS replacement ratio and curing age was observed with respect to concrete porosity. At 7 days of curing, the porosities of specimens with LS replacement ratios of 10%, 15%, 20%, 25%, and 30% were 7.104%, 7.179%, 7.60%, 8.186%, and 7.580%, respectively, whereas the control group (0% LS) exhibited a porosity of 9.151%. After 28 days of curing, the porosities decreased to 5.028% (control), 5.028%, 6.014%, 5.733%, 6.582%, and 6.672% for the respective groups.

The variation in porosity reflects a competitive interplay between the physical filling effect and the pozzolanic chemical reactivity of LS. On one hand, the fine particles of LS act as micro-fillers, occupying intergranular voids and refining the pore structure, which contributes to porosity reduction. On the other hand, the pozzolanic reaction between the active SiO₂ and Al₂O₃ in LS and the calcium hydroxide generated during cement hydration produces additional C-S-(A)-H gels. These secondary hydration products further densify the microstructure over time. However, excessive LS replacement reduces the cement content, thereby limiting the availability of calcium hydroxide required for the pozzolanic reaction. Consequently, at higher replacement levels, the positive effect of pozzolanic reaction is compromised, leading to less effective pore refinement or even increased porosity. This competitive mechanism, wherein the beneficial effects of filling and pozzolanic reaction are counterbalanced by the dilution of cementitious components, accounts for the nonlinear relationship between LS content and concrete porosity^51^.

Dual techniques were used to optimize the porosity structure when the incorporation of LS was less than 20%. Capillary pores were preferentially filled by finer LS particles (physical filling effect)^52,53^, which decreased the overall pore volume. While C-S-H gel formation was hindered by early-stage Ca(OH)₂ deficit, microcrack propagation was successfully suppressed by the concentrated reaction products at interfacial transition zones (ITZ). Synergistic physic-chemical effects peaked at the ideal 20% replacement level, resulting in a 10.46% reduction in porosity at 28 d when compared to the control group. Nevertheless, higher than 20% LS integration caused secondary ettringite (AFt) nucleation by accelerating the rates of SO₄²⁻ dissolution. At 28 d of curing, this procedure produced localized expansion stresses that led to an unusual increase in porosity (6.672% vs. control 6.403%).

According to the experimental findings, at low levels of LS incorporation, LS mainly fills macro-pore structures in concrete by acting as a fine aggregate through micro-aggregate effects. Pozzolanic reactions take place in alkaline settings as the LS content rises. Significant mixture proportioning effects are confirmed by the synergistic interplay between pozzolanic processes and filling effects, which maximizes compressive strength at 20% LS integration while minimizing porosity. But as the LS content rises beyond 20%, it causes chemical interactions between the LS’s SO₄²⁻ and hydration products, resulting in expansive phases such ettringite (Ca₆Al₂(SO₄)₃(OH)₁₂·26 H₂O). This causes volumetric expansion, encourages the spread of internal cracks, and gradually raises porosity.

**Fig. 7:**
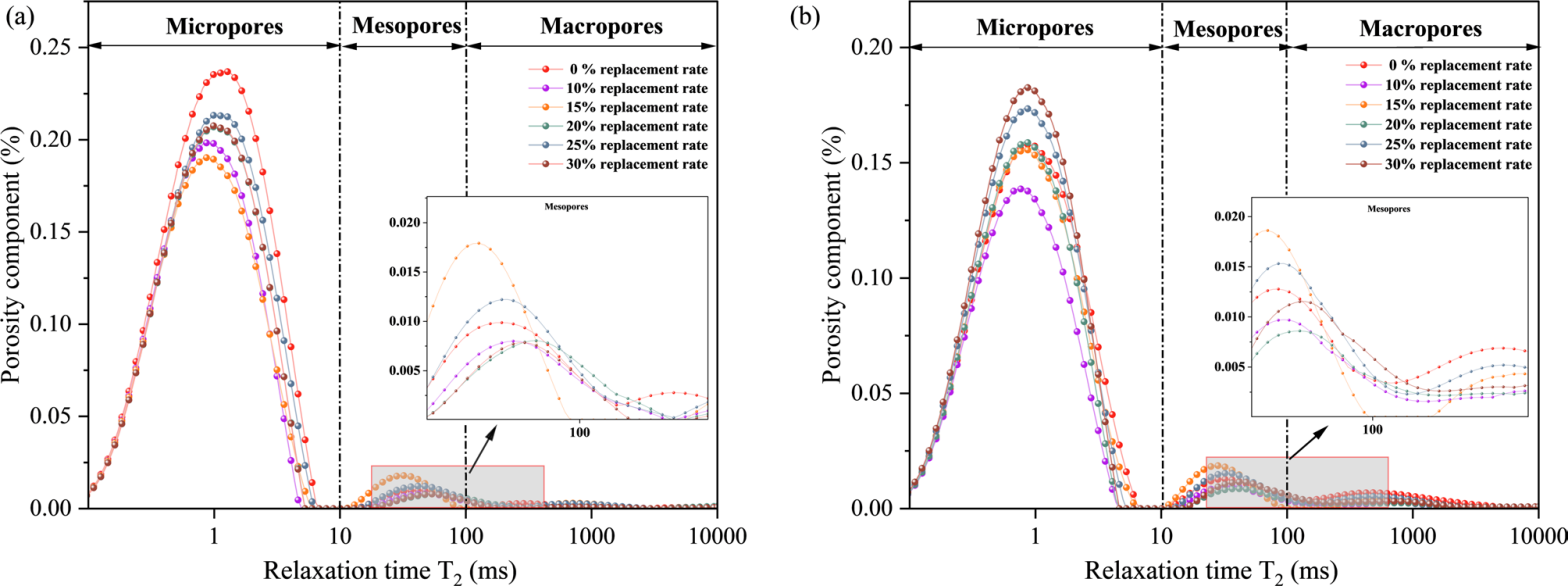
T2 distribution of LS concrete with different admixture contents. (**a**) 7d, (**b**) 28d.

**Fig. 8:**
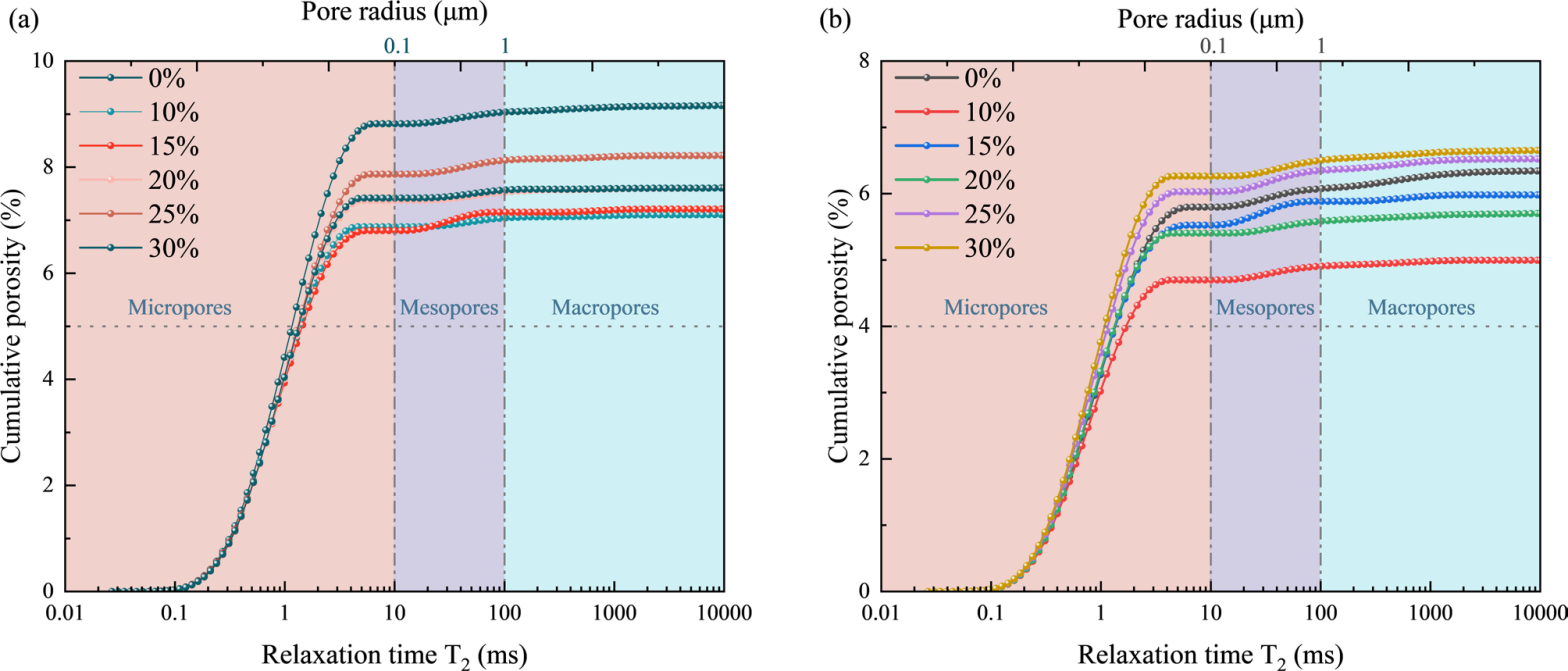
Cumulative pore size diagrams of LS concrete with different admixture contents. (**a**) 7d, (**b**) 28d.

**Fig. 9:**
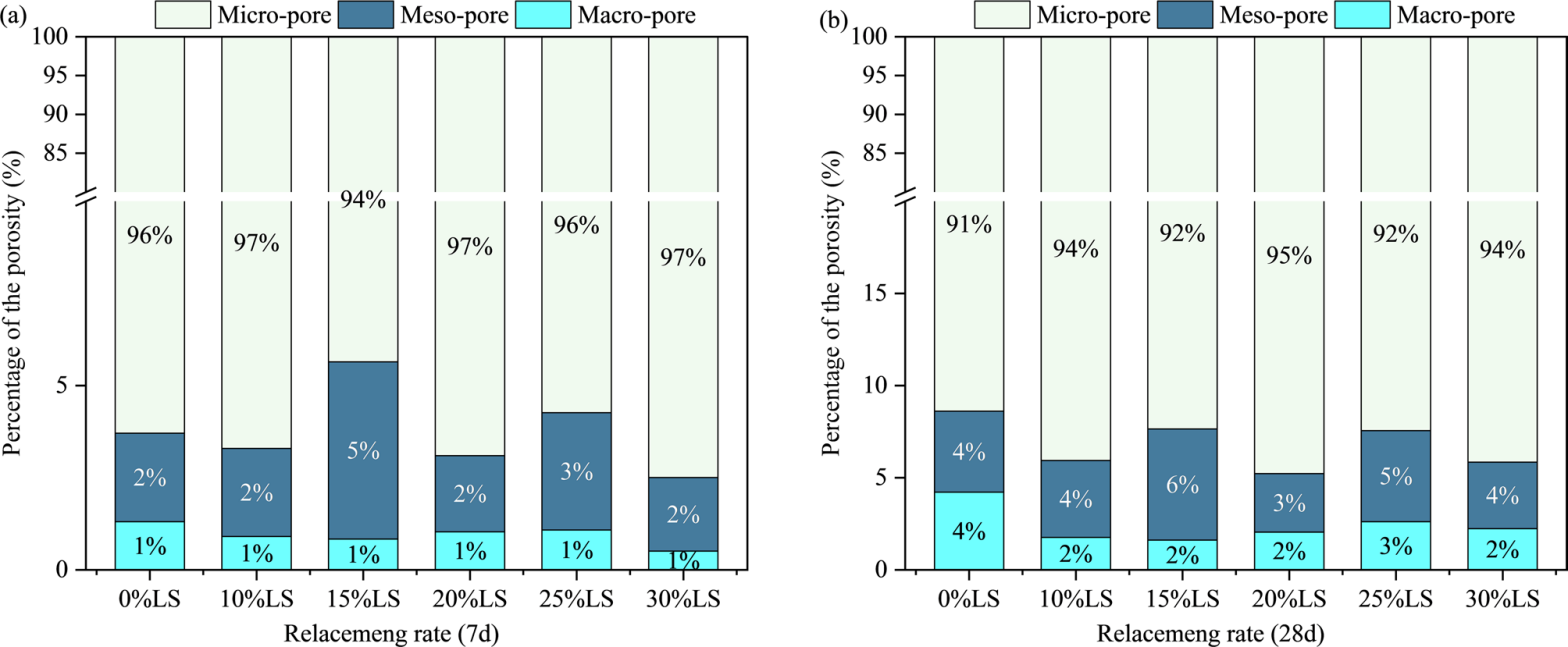
The quantitative statistical results of the pore size fraction. (**a**) 7d, (**b**) 28d.

LS incorporation ratios (0%, 10%, 15%, 20%, 25%, and 30%) and curing ages (7 d and 28 d) on concrete pore hierarchy were analyzed to examine the multi-scale correlation mechanism between pore structure and compressive strength. Figure 7 displays the T₂ relaxation time distribution curves for seven d-cured specimens, and Fig. 8 displays the T₂ relaxation time distribution curves for twenty-eight d-cured specimens. Figure 9 shows the quantitative statistical results of the pore size fractions.

The micro-pore proportion showed a “rise-decline-rise” trend with increasing LS incorporation at 7 d curing, as shown by the experimental results in Figs. 7, 8 and 9. The analysis of pore structure evolution from 7 to 28 days reveals a critical distinction between the control and the lithium slag-modified samples, particularly regarding the development of coarse porosity. In the control group, the proportion of large pores exhibited a notable increase from 1.31% at 7 days to 4.23% at 28 days, indicating a coarsening of the pore structure during this curing period. This phenomenon is often associated with the continued, potentially less dense, growth of hydration products or the development of micro-cracks in the absence of supplementary cementitious materials. In stark contrast, all mixtures incorporating alkali-activated and mechanically activated lithium slag demonstrated a significant mitigation of this trend. For instance, even at a 30% replacement level, the large pore content remained markedly lower at 2.24% after 28 days, representing an approximately 47% reduction compared to the control. This suppression of coarse pore development underscores the efficacy of the dual activation treatment in refining the microstructure. Consequently, the modified systems exhibit a superior pore structure characterized not only by a higher volume of harmless and less harmful pores but also by a enhanced stability against the formation of detrimental large pores over time, which is pivotal for long-term durability and mechanical performance.

The continuous pore-filling effect is primarily attributed to the persistent formation of C-S-H gel through the pozzolanic reaction. Under alkaline conditions, a synergistic interaction occurs between this pozzolanic process and the alkali-activated cementitious products, which not only contributes to pore structure refinement but also effectively mitigates internal expansion and suppresses the propagation of micro-cracks. However, when the incorporation level of lithium slag (LS) exceeds 20%, the synthesis of ettringite (AFt) is delayed due to secondary reactions involving sulfate ions (SO₄²⁻) and the prevailing hydration products. The resultant localized expansive stress promotes an increase in the proportion of macro-pores and facilitates the development of macroscopic cracks^54,55^.

### TG-DTG

**Fig. 10:**
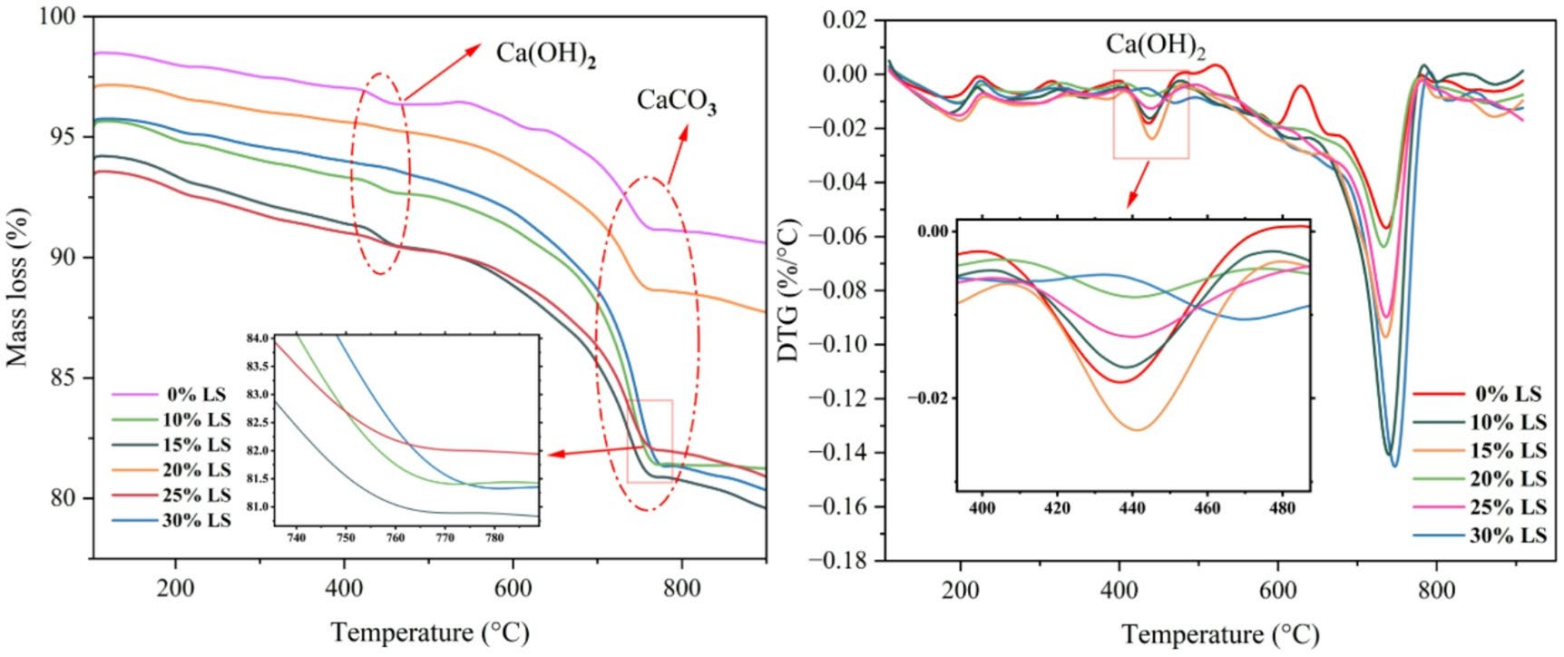
TG diagram and DTG result of LS concrete.

TG-DTA of concrete specimens with different LS replacement ratios was used to examine the impact of LS incorporation on hydration products in cementitious materials. Figure 10 displays the findings of the statistical study.

LS presence significantly affects the hydration reaction processes and product compositions, as illustrated in Fig. 10. The control group (0% LS) showed a clear Ca(OH)₂ dehydroxylation peak in the 400–500 °C temperature range^56,57^. This peak intensity progressively decreased with increased LS content. The 20% LS group had the lowest mass loss rate. This finding demonstrates that the 20% replacement level was where pozzolanic reactions transpired most complete.

Ca(OH)₂ consumption was lower in the low-dosage groups (10%-15% LS) than in the control group, indicating insufficient activation of LS pozzolanic reaction at these incorporation levels. CaCO₃ decomposition peaks were present in all specimens in the 700–800 °C temperature range. In contrast to the control and 30% LS groups, the 20% LS group displayed a noticeably lower mass loss rate. More thorough pozzolanic reactions that reduced the carbonation potential of free Ca^2+^ ions are thought to be the cause of these phenomena^58,59^.

According to microstructural examination, higher incorporation groups produced micro-sized calcite aggregates because of incomplete reactions, whereas the 20% LS group had uniformly distributed nano-sized CaCO₃ particles. Reaction efficiency dropped after 20% LS inclusion due to localized excessive alkalinity and particle agglomeration. These findings show that in cementitious systems, LS inclusion of about 20% best stimulates hydration reactions and maximizes product composition.

### SEM-EDS

**Fig. 11:**
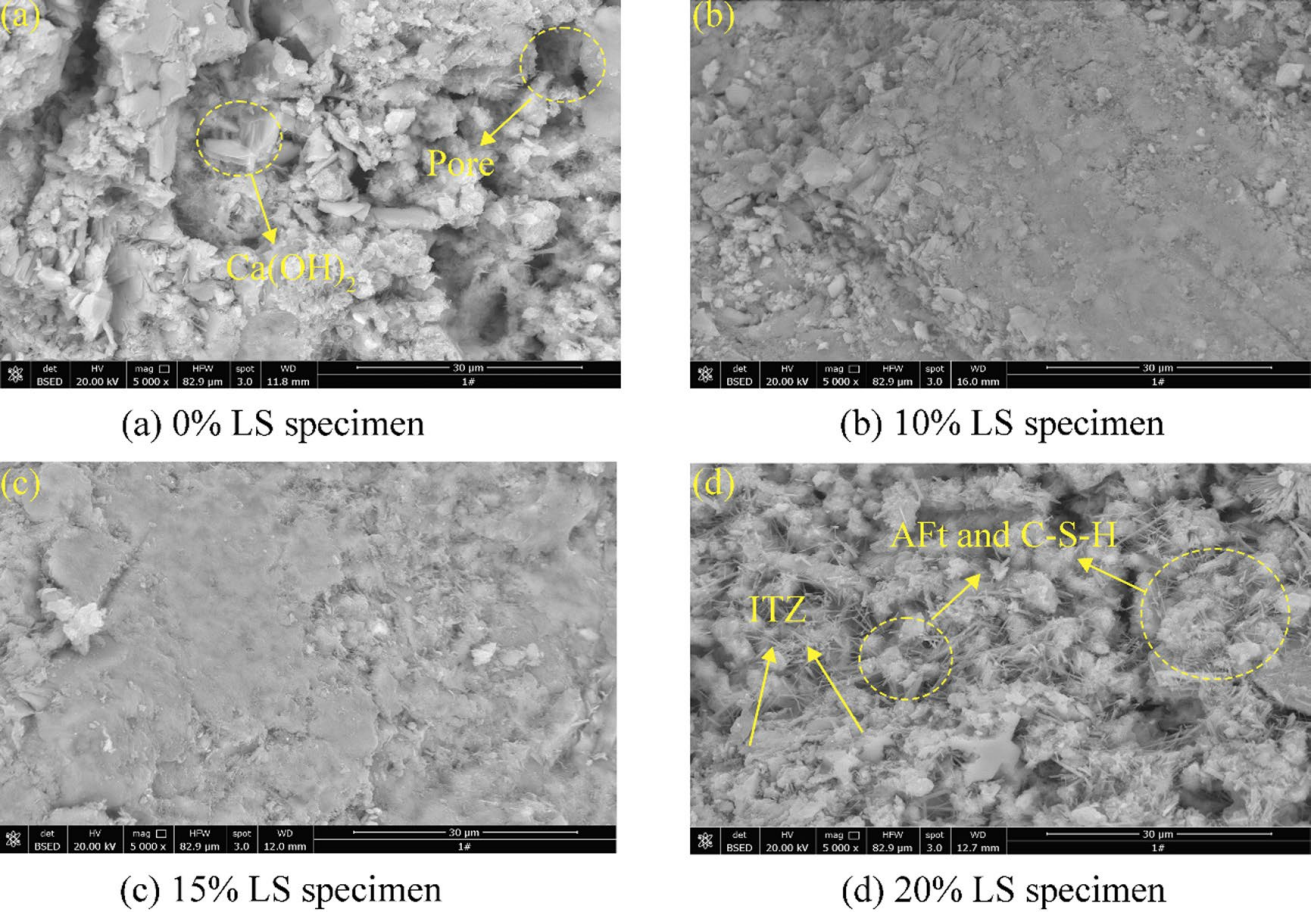
SEM diagram of LS concrete with different dosage.

SEM was used to examine the evolution of hydration products and microstructure in concrete specimens with different ratios of LS content (0%, 10%, 15%, and 20%), as illustrated in Fig. 11.

As shown in Fig. 11, both the control and low-dosage groups (10–15% LS) showed characteristic hexagonal plate-like Ca(OH)₂ crystals. These crystals matched well with the substantial mass loss peaks at 400–500 °C that were found by TGA^60,61^. Acicular ettringite (AFt) crystals interlaced with high-density amorphous C-S-H gels created a three-dimensional network structure in the 20% LS specimen, which had distinctive microstructural characteristics.

The SO₃ components of LS activated the sulfate under high pH (pH = 12) conditions, causing the structural alterations that were seen. Ettringite (AFt) crystals and C-S-H gels co-developed more readily in this alkaline environment^60^. This dual-phase hydration method successfully reduced porosity in ITZ, according to microstructural measurement. Better mechanical performance was a direct result of the denser microstructure. However, growth became troublesome when LS additions exceeded 20%. Detrimental volumetric changes resulted from the excess sulfate ions (SO₄²⁻) promoting delayed ettringite formation.

**Fig. 12:**
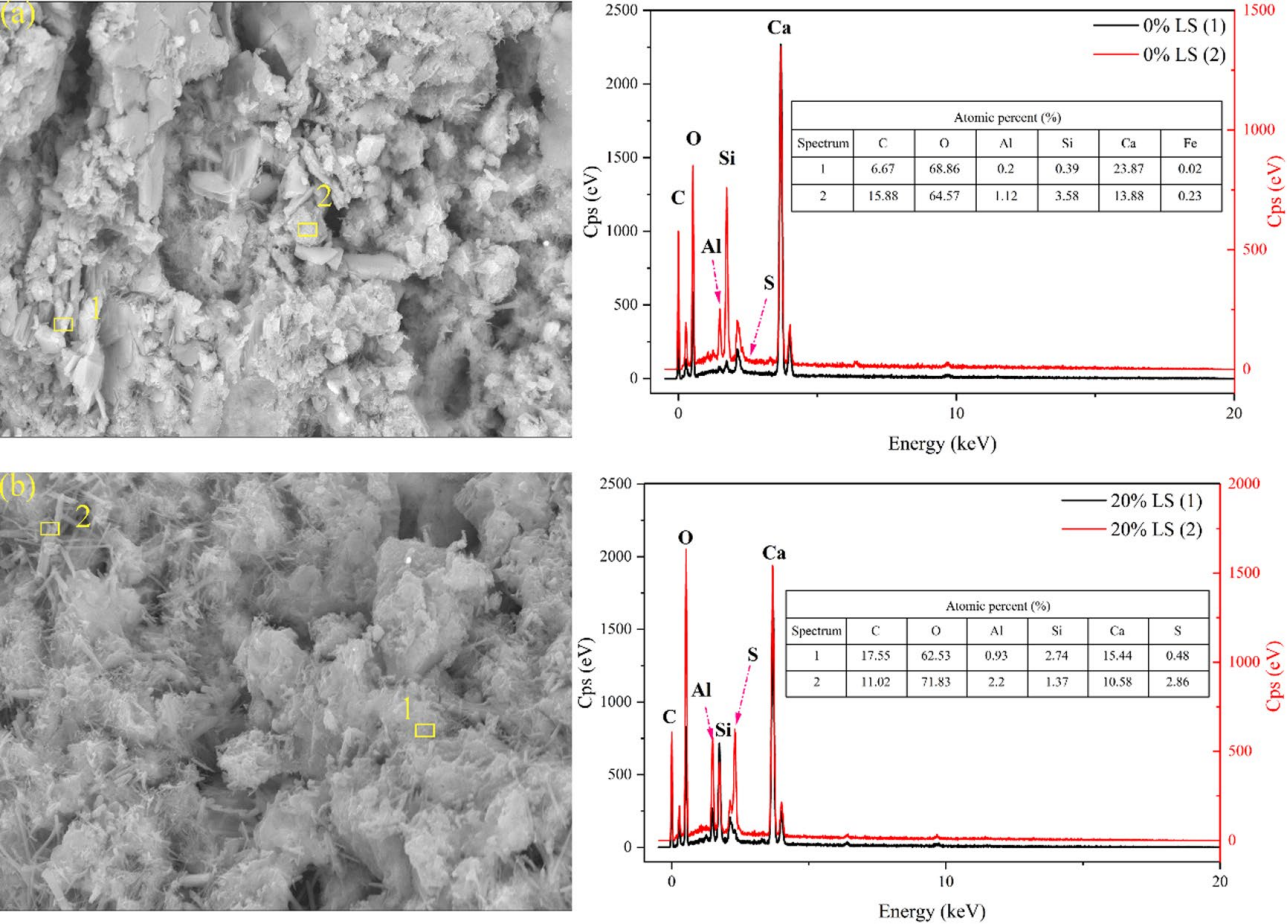
EDS diagram of partial LS concrete. (**a**) 0% LS; (**b**) 20% LS.

Using EDS, the differences in hydration products in concrete with different ratios of LS content were examined, as illustrated in Fig. 12. The acicular ettringite (AFt) crystal area (Spot 2) in the 20% LS specimen showed clear Ca, S, Al, and O typical peaks^61^.

Combining thermal analysis results with the notable detection of carbon signals (~ 0.28 keV) in this region suggests that AFt may partially convert to AFm during subsequent curing stages^62^. Ca(OH)₂ crystals were confirmed by the fact that Spot 1 in control specimens only displayed Ca and O signals. While the elemental composition at Spot 2 was in line with standard C-S-H gels, the Al content was noticeably lower than in the LS-modified specimens. These results provide important insights into the mechanisms underlying the strength development and durability performance of LS-incorporated concrete by microscopically validating that LS incorporation not only modifies the chemistry of hydration products but also encourages the creation of sulfoaluminate phases.

### FTIR

**Fig. 13:**
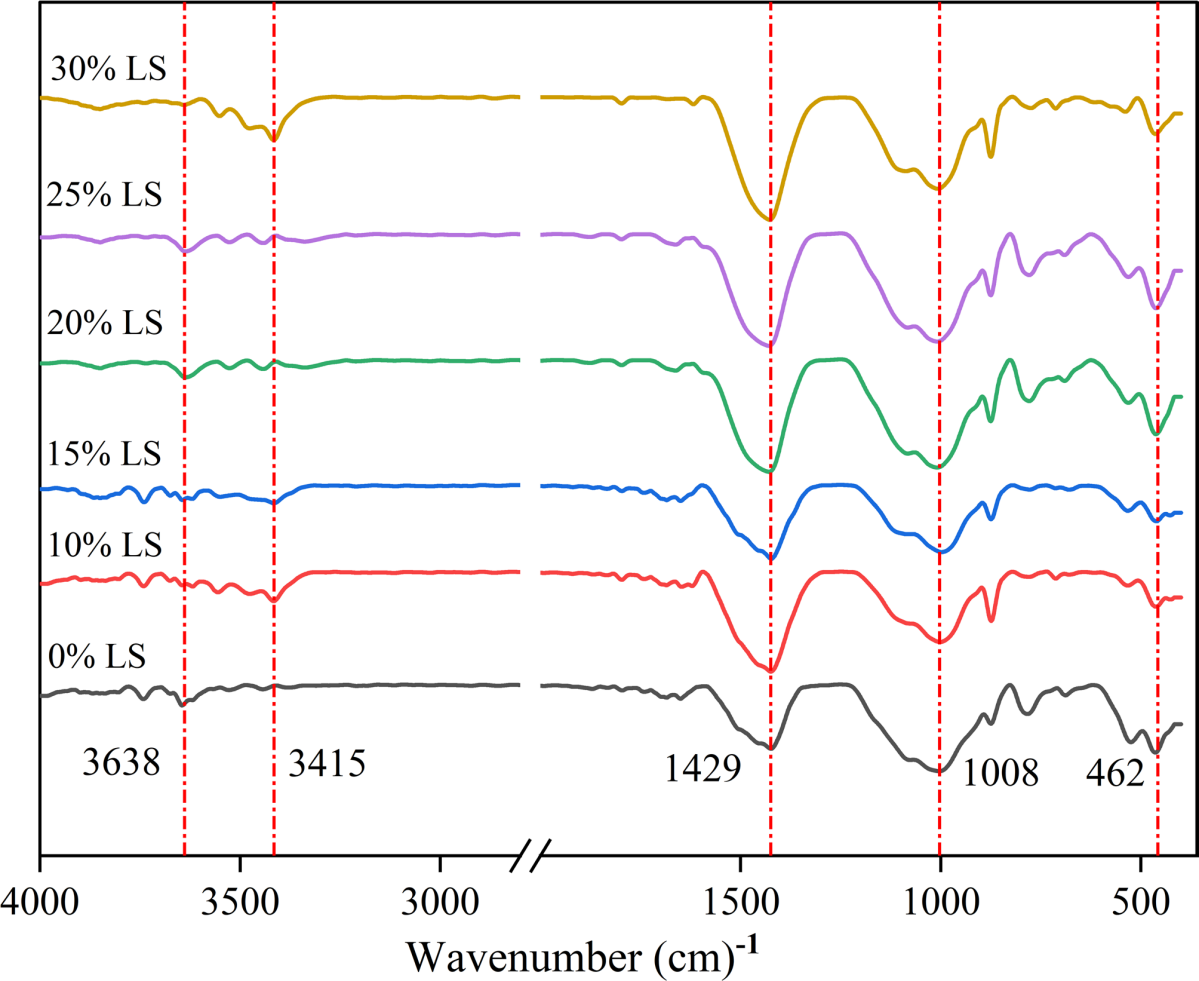
FTIR spectra of LS concrete with different dosage.

FTIR spectroscopy elucidated the regulatory effects of LS on cement hydration pathways (Fig. 13). The prominent O-H stretching band at 3400 cm⁻¹ in LS-modified specimens demonstrated significantly enhanced water adsorption capacity. This phenomenon directly correlates with the ultrafine particle characteristics of LS, as evidenced by its high specific surface area^47,63^. In contrast, control specimens exhibited markedly weaker absorption in this spectral region. A progressive intensity increases at 1429 cm⁻¹ was observed with higher LS incorporation rates. Spectral deconvolution identified this as resulting from two concurrent processes: intrinsic calcite presents in LS, and alkali-catalyzed carbonation reactions^15^. These findings demonstrate LS’s dual role in modifying both initial hydration kinetics and long-term phase evolution. LS influences cement hydration through multiple pathways: by altering hydration reaction during early stages, and by participating in subsequent carbonate formation processes. This multiscale modification mechanism explains the observed improvements in cementitious system performance.

Significant differences in the O-Si-O bending vibration at 462 cm⁻¹ were found by FTIR analysis, with the 10% and 15% LS groups exhibiting significantly lower intensity than the 20% LS specimens. This finding was supported by NMR quantitative analysis, which showed lower degrees of silicate polymerization in the lower dosage groups, confirming their limited pozzolanic reactivity. The O-H bending vibration at 3638 cm⁻¹ exhibited an inverse relationship with compressive strength. Notably, while the 30% LS group displayed weaker Ca(OH)₂ signals than the control, it failed to show mechanical improvement. This unexpected result can be explained by the counteracting effects of delayed ettringite formation caused by excessive SO₄²⁻, which negated the potential benefits of pore structure refinement.

To optimize hydration kinetics in concrete systems with LS incorporated, these molecular-level characterizations created significant correlations with macroscopic mechanical behavior. To attain optimal performance while avoiding harmful expansion effects, the results emphasize the significance of balanced LS inclusion^62,63^.

## Discussion

**Fig. 14:**
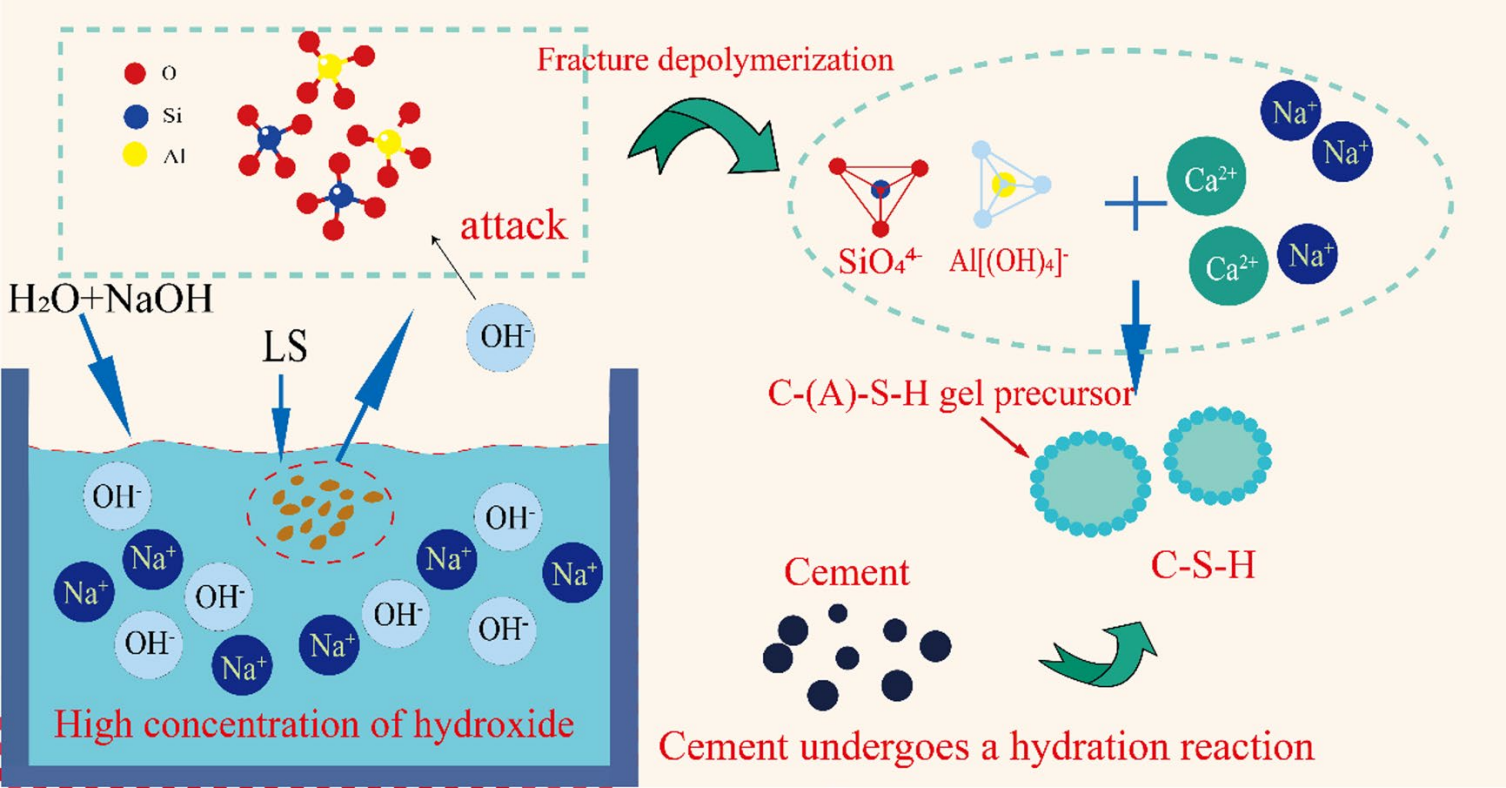
The reaction mechanism diagram of LS.

Cementitious material hydration evolves dynamically throughout several stages. Through interface dissolution-precipitation mechanisms, tricalcium silicate (C₃S) quickly forms microscale C-S-H gel networks in the initial stage, creating the early-strength framework^64^. The microscopic reaction mechanism of alkali-mechanically activated LS concrete is shown in Fig. 14. As a SCM, LS improves the reaction kinetics throughout this procedure in two ways. On the one hand, its high specific surface area micron-scale particles provide many nucleation sites for heterogeneous C-S-H growth. However, when used in place of some of the cement clinker, LS provides suitable surfaces for product precipitation and more room for hydrate formation. The formation of dense microstructures is encouraged by this dual process^56^. While the reactive surfaces promote directed formation of crystalline phases, the increased spatial accommodation allows for a more uniform distribution of hydration products. Together, these actions boost both the speed and quality of microstructure formation during cement hydration.

Diffusion-controlled processes continuously consume unreacted dicalcium silicate (C₂S) and reactive Si-Al phases from LS throughout the hydration process in prolonged curing stages. Under strongly alkaline circumstances, amorphous SiO₂ in LS experiences bond cleavage and dissociation, producing SiO_3_^2^⁻ monomers that join Ca²⁺ to produce highly polymerized C-S-H. Nevertheless, when LS integration exceeds 20%, surplus SO₄²⁻ and calcium aluminate phases react, resulting in the formation of delayed ettringite (AFt). In the ITZ, the resulting expansive pressures cause microcracks that negate the advantageous effects of pozzolanic processes and hence lower compressive strength.

The strength reduction at LS replacement levels above 20% is primarily attributed to sulfate-induced expansion, supported by the following experimental evidence. (1) TG-DTG analysis shows increased mass loss in the 100–200 °C region corresponding to AFt dehydration at 25% and 30% LS. (2) SEM images reveal microcracks in the 25% and 30% specimens. (3) NMR porosity data shows anomalous increase in large pore fraction (> 100 nm) at these high replacement levels. The contribution of ASR is hypothesized based on the high reactive silica content and alkaline conditions (pH = 12), but direct evidence (petrographic examination for alkali-silica gel) was not obtained in this study. Particle agglomeration at high LS content is inferred from the reduced pozzolanic efficiency indicated by lower Ca(OH)₂ consumption in TG analysis, but direct particle distribution measurements in hardened paste were not performed.

Under alkaline conditions (pH = 12), amorphous aluminosilicates in LS undergo significant dissolution-repolymerization processes. Si-O bonds are cleaved and depolymerized under high OH⁻ concentrations, generating soluble silicate species (e.g., SiO_3_²⁻, Eq. (2)). Concurrently, Al-O bonds undergo coordination restructuring to form stabilized aluminate species (e.g., Al(OH)₄⁻, Eq. (3))^8,65^.

2 $$Si{O_2} + 2O{H^ - } \to Si{O_3}^{2 - } + {H_2}O$$

3 $$A{l_2}{O_3} + 2O{H^ - } + 3{H_2}O \to 2Al{\left( {OH} \right)_4}^ -$$

In cement systems treated with LS, the hydration process demonstrates a multi-ion synergistic mechanism. Under very alkaline circumstances, dissolved silicate (SiO_3_^2^⁻) and aluminate (Al(OH)₄⁻) species coordinate with Ca²⁺ and Na⁺ ions to form core-shell structured C-(A)-S-H gel precursors. As spherical nanoparticles, their precursors preferentially nucleate at ≡ Si-O⁻ active sites on LS surfaces. Ca-O-Si covalent bonding then establishes chemical integration with the matrix.

The dissolved silicate (SiO_3_^2^⁻) and aluminate (Al(OH)₄⁻) ions are coordinated with alkaline cations (e.g., Na⁺, K⁺, Ca²⁺) in solution to form gelation precursors, which serve as nucleation sites for C-(A)-S-H gels. As shown in Fig. 10, flocculent C-S-H predominantly exhibits spherical attachment morphology. This occurs through activation of ≡ Si-O⁻ and ≡ Al-O⁻ sites on slag surfaces by the Na⁺/OH⁻ environment, enabling chemical grafting with C-S-H via corresponding bonds to form dense core-shell structures. The alkaline environment significantly enhances Ca²⁺ dissolution rates from C₃S surfaces (Eq. 4), driving liquid-phase Ca²⁺ concentration to portlandite (CH) supersaturation within 12 h. During curing, CH continuously reacts with SiO₄⁴⁻ ions released from LS, further generating hydration products.

4 $${C_3}S + {H_2}O \to C{a^{2 + }} + O{H^ - } + C - S - H$$

The hydration reaction of C₃S (Eq. 4) is modulated by Na⁺ concentration in pore solution. Elevated Na⁺ levels induce ionic strength effects that reduce Ca(OH)₂ solubility, triggering rapid CH crystallization^66^. Simultaneously, dissolved SO₄²⁻ combines with aluminate species to form acicular ettringite (Eq. 5), which grows preferentially along C-S-H/LS interfaces, enhancing ITZ bonding strength through mechanical interlocking. This multiphase synergistic effect reaches optimal performance at the 20% incorporation level.

5 $$6C{a^{2 + }} + 2Al{\left( {OH} \right)^{4 - }} + 3S{O_4}^{2 - } + 32{H_2}O \to 3CaO \cdot A{l_2}{O_3} \cdot 3CaS{O_4} \cdot 32{H_2}O$$

Under highly alkaline conditions, expansive strain is induced in concrete by sodium silicate colloids generated through alkali-silica reaction (ASR), resulting in increased microcrack density^54,55^. This damage mechanism predominantly localizes at ITZ, leading to compressive strength degradation in LS-incorporated concrete. Compared with ordinary Portland cement (OPC) systems, LS-modified cement exhibits elevated hydration heat release that potentially exacerbates pore structure development during early curing stages. However, the physical packing effects of LS particles effectively counterbalance this inherent drawback through optimized particle gradation and pore refinement, ultimately enhancing mechanical performance.

**Table 3:** Comparative analysis with key previous studies.

Study	Optimum LS%	Activation Method	Key Characterizatio
He et al.^20,22,23^	20%	Raw LS	Strength, SEM
Amin et al.^28^	40% (180 d)	Raw LS	Porosity, transport
Zhang et al.^24^	20%	Raw LS	XRD, SEM
This study	20%	Mechano-alkali synergy	NMR, TG-DTG, FTIR, SEM-EDS

As shown in the Table 3, while previous studies have identified the 20% optimum through empirical strength testing, our study is the first to provide comprehensive mechanistic quantification of the synergistic activation effects using multi-scale characterization techniques.

## Conclusion

This study systematically investigated the influence mechanisms of activated LS replacement ratios (10–30%) on the mechanical properties and microstructure of cement-based composites. The key findings are summarized as follows:

1. Mechanical performance exhibited significant nonlinear dependence on replacement ratios. The 20% replacement group demonstrated optimal compressive strength, while an anomalous strength value observed in the 15% group revealed the critical proportion effect in cementitious material systems.
2. Multi-scale characterization verified that activated LS optimizes pore structure through a synergistic mechanism combining chemical activation and physical filling: NMR porosity analysis showed that the 10% replacement group had the lowest porosity, while SEM microstructural analysis showed that the 20% group had the most compact hydration product network.
3. Pore structure evolution showed that LS-modified groups showed a significant reduction in harmful pore proportion with extended curing to 28 d. Microstructural experiments confirmed that LS’s pozzolanic activity effectively promotes secondary hydration reactions.

## Data Availability

The datasets used and/or analyzed during the current study are available from the corresponding author on reasonable request.

## References

[1] Hu, R., Cai, T. & Xu, W. Exploring the technology changes of new energy vehicles in China: Evolution and trends. *Comput. Ind. Eng.* **191**, 110178 (2024).

[2] Karrech, A., Azadi, M. R., Elchalakani, M., Shahin, M. A. & Seibi A. C. A review on methods for liberating lithium from pegmatities. *Miner. Eng.* **145**, 106085 (2020).

[3] Jiang, M. et al. Microwave-enhanced sulfate roasting for lithium extraction from lepidolite: A comprehensive study. *J. Clean. Prod.* **434**, 140248 (2024).

[4] Gu, T. et al. The formation, characteristics, and resource utilization of lithium slag. *Constr. Build. Mater.* **432**, 136648 (2024). Review.

[5] Liu, C., Lin, J., Cao, H., Zhang, Y. & Sun, Z. Recycling of spent lithium-ion batteries in view of lithium recovery: A critical review. *J. Clean. Prod.* **228**, 801–813 (2019).

[6] Yiren, W., Dongmin, W., Yong, C., Dapeng, Z. & Ze, L. Micro-morphology and phase composition of lithium slag from lithium carbonate production by sulphuric acid process. *Constr. Build. Mater.* **203**, 304–313 (2019).

[7] Yang, B. et al. Recycling lithium slag into eco-friendly ultra-high performance concrete: Hydration process, microstructure development, and environmental benefits. *J. Building Eng.* **91**, 109563 (2024).

[8] Liu, Z. et al. A green route to sustainable alkali-activated materials by heat and chemical activation of lithium slag. *J. Clean. Prod.* **225**, 1184–1193 (2019).

[9] Chen, X. et al. Effect of dosage of lithium slag powder on the microstructure and hydration of ultra-low water-to-binder ratio lithium slag-based concrete prepared by microwave pre-curing. *Constr. Build. Mater.* **514**, 145421 (2026).

[10] Mahmoud, A. A. et al. Evaluation of rice husk biochar influence as a partial cement replacement material on the physical, mechanical, microstructural, and radiation shielding properties of ordinary concrete. *Sci. Rep.* **15**, 27229 (2025). 40715340 10.1038/s41598-025-11987-8 PMC12297212

[11] Elbauomy, A. K. et al. Mechanical, microstructural, and radiation shielding characteristics of sustainable high-strength concrete incorporating recycled wastes blended powders. *Sci. Rep.* **15**, 40398 (2025). 41253916 10.1038/s41598-025-23824-z PMC12627603

[12] Mahmoud, A. A. et al. Elevated temperature effects on the compressive strength and radiation shielding capability of waste granite and marble concrete. *Eur. Phys. J. Plus*. **140**, 302 (2025).

[13] Abouelnour, M. A. et al. Valorization of nano additives effects on the physical, mechanical and radiation shielding properties of high strength concrete. *Sci. Rep.* **15**, 14440 (2025). 40281078 10.1038/s41598-025-99126-1 PMC12032053

[14] Li, J., Lian, P., Huang, S. & Huang, L. Recycling of lithium slag extracted from lithium mica by preparing white Portland cement. *J. Environ. Manage.* **265**, 110551 (2020). 32275252 10.1016/j.jenvman.2020.110551

[15] Zhai, Q., Liu, R., Song, Y., Mao, Z. & Sun, W. A novel technology for lithium extraction through low-temperature synergistic roasting of α-spodumene with lepidolite. *Sep. Purif. Technol.* **355**, 129667 (2025).

[16] Zhang, Y., Ma, B., Lv, Y., Wang, C. & Chen, Y. An effective method for directly extracting lithium from α-spodumene by activated roasting and sulfuric acid leaching. *J. Ind. Eng. Chem.* **122**, 540–550 (2023).

[17] Wang, F., Yang, M., Yang, Y. & Tian, Y. Synergistic leaching of lithium from clay-type lithium ore using sulfuric acid and oxalic acid. *Appl. Clay Sci.* **262**, 107623 (2024).

[18] Yan, Q. et al. Extraction of lithium from lepidolite by sulfation roasting and water leaching. *Int. J. Miner. Process.* **110–111**, 1–5 (2012).

[19] Li, J. & Huang, S. Recycling of lithium slag as a green admixture for white reactive powder concrete. *J. Mater. Cycles Waste Manag*. **22**, 1818–1827 (2020).

[20] He, Y., Kang, Q., Lan, M. & Peng, H. Mechanism and assessment of the pozzolanic activity of melting-quenching lithium slag modified with MgO. *Constr. Build. Mater.* **363**, 129692 (2023).

[21] Li, W. & Fall, M. Sulphate effect on the early age strength and self-desiccation of cemented paste backfill. *Constr. Build. Mater.* **106**, 296–304 (2016).

[22] He, Z., Li, L. & Du, S. Mechanical properties, drying shrinkage, and creep of concrete containing lithium slag. *Constr. Build. Mater.* **147**, 296–304 (2017).

[23] He, Z., Du, S. & Chen, D. Microstructure of ultra high performance concrete containing lithium slag. *J. Hazard. Mater.* **353**, 35–43 (2018). 29631045 10.1016/j.jhazmat.2018.03.063

[24] Zhang, Y., Yang, B., Gu, X., Han, D. & Wang, Q. Improving the performance of ultra-high performance concrete containing lithium slag by incorporating limestone powder. *J. Building Eng.* **72**, 106610 (2023).

[25] Javed, U., Shaikh, F. U. A. & Sarker, P. K. Microstructural investigation of lithium slag geopolymer pastes containing silica fume and fly ash as additive chemical modifiers. *Cem. Concr. Compos.* **134**, 104736 (2022).

[26] Luo, X. et al. A technique for preparing one-part geopolymers by activating alkali-fused lithium slag with solid sodium silicate. *Constr. Build. Mater.* **435**, 136817 (2024).

[27] Luukkonen, T., Abdollahnejad, Z., Yliniemi, J., Kinnunen, P. & Illikainen, M. One-part alkali-activated materials: A review. *Cem. Concr. Res.* **103**, 21–34 (2018).

[28] Amin, M. T. E., Sarker, P. K. & Shaikh, F. U. A. Transport properties of concrete containing lithium slag. *Constr. Build. Mater.* **416**, 135073 (2024).

[29] Rahman, S. A., Shaikh, F. U. A. & Sarker, P. K. A comprehensive review of properties of concrete containing lithium refinery residue as partial replacement of cement. *Constr. Build. Mater.* **328**, 127053 (2022).

[30] Gao, S. et al. Synergistic effects of fly ash-cement slurry and CO2 mineralization on coal gangue aggregate and its concrete properties. *Constr. Build. Mater.* **465**, 140225 (2025).

[31] Moussadik, A., El Fadili, H., Saadi, M. & Diouri, A. Lightweight aerated concrete based on activated powders of coal gangue and fly ash. *Constr. Build. Mater.* **417**, 135333 (2024).

[32] Alanyali, H., Çöl, M., Yilmaz, M. & Karagöz, Ş. Concrete Produced by Steel-Making Slag (Basic Oxygen Furnace) Addition in Portland Cement. *Int. J. Appl. Ceramic Tech.* **6**, 736–748 (2009).

[33] Li, Y., Liu, F., Yu, F. & Du, T. A review of the application of steel slag in concrete. *Structures* **63**, 106352 (2024).

[34] Pushpakumara, B. H. J. & Bandara, P. M. K. N. Evaluating the effectiveness of copper slag waste as a fine aggregate in concrete. *Constr. Build. Mater.* **475**, 141046 (2025).

[35] Meng, L. Y., Wang, Y. S., Sun, F., Lin, R. & Wang, X. Y. An integrated strength-carbon emissions-total cost model for silica fume concrete. *Case Stud. Constr. Mater.* **22**, e04327 (2025).

[36] Song, S. & Jennings, H. M. Pore solution chemistry of alkali-activated ground granulated blast-furnace slag. *Cem. Concr. Res.* **29**, 159–170 (1999).

[37] Javed, U., Ahmed Shaikh, U., Kumar Sarker, P. & F. & Microstructural investigation of thermo-mechanically processed lithium slag for geopolymer precursor using various characterization techniques. *Constr. Build. Mater.* **342**, 127952 (2022).

[38] Javed, U., Shaikh, F. U. A. & Sarker, P. K. A comprehensive micro-nano investigative approach to study the development of aluminosilicate gel in binary blends of lithium slag geopolymer. *Cem. Concr. Compos.* **145**, 105338 (2024).

[39] Zhou, X., Tang, Z., Zheng, Y., Zhang, Y. & Wu, F. Research on the properties and mechanism of a fiber-reinforced alkali-activated lithium slag artificial lightweight aggregate. *Constr. Build. Mater.* **472**, 140866 (2025).

[40] Ye, J. et al. The leaching model and leaching kinetics of lithium slag in alkaline solution. *Constr. Build. Mater.* **432**, 136642 (2024).

[41] Li, J. et al. The chemistry and structure of calcium (alumino) silicate hydrate: A study by XANES, ptychographic imaging, and wide- and small-angle scattering. *Cem. Concr. Res.* **115**, 367–378 (2019).

[42] Zhang, Y., Zhang, L., Wang, Q., Han, D. & Li, Z. Iron ore tailings, phosphate slags, and lithium slags as ternary supplementary cementitious materials for concrete: Study on compression strength and microstructure. *Mater. Today Commun.* **36**, 106644 (2023).

[43] Wang, S. et al. Assessment of lithium slag as a supplementary siliceous material in autoclaved aerated concrete: Physical properties and hydration characteristics. *Constr. Build. Mater.* **442**, 137621 (2024).

[44] Kunther, W., Lothenbach, B. & Skibsted, J. Influence of the Ca/Si ratio of the C–S–H phase on the interaction with sulfate ions and its impact on the ettringite crystallization pressure. *Cem. Concr. Res.* **69**, 37–49 (2015).

[45] Luo, Q. et al. Lithium slag-based geopolymer synthesized with hybrid solid activators. *Constr. Build. Mater.* **365**, 130070 (2023).

[46] Alexander, K. M., Wardlaw, J. & Ivanusec, I. The influence of SO3 content of portland cement on the creep and other physical properties of concrete. *Cem. Concr. Res.* **9**, 451–459 (1979).

[47] Wang, Y., Wang, D., Zheng, Y., Hua, K. & Liu, J. Thermal activation mechanism and activity evaluation of lithium slag: Insights from simulated hydration. *Constr. Build. Mater.* **411**, 134615 (2024).

[48] Tan, H. et al. Utilization of lithium slag by wet-grinding process to improve the early strength of sulphoaluminate cement paste. *J. Clean. Prod.* **205**, 536–551 (2018).

[49] Qiu, Y., Wu, D., Yan, L. & Zhou, Y. Recycling of spodumene slag: preparation of green polymer composites. *RSC Adv.* **6**, 36942–36953 (2016).

[50] Zhang, K. et al. Durability, ecological benefits and carbon emission effects on eco-friendly solid waste concrete containing lithium slag. *Case Stud. Constr. Mater.* **24**, e05845 (2026).

[51] Zhao, H., Huang, H., Tang, J., Yao, C. & Gao, X. Thermal activation of lithium slag and its performance in ultra-high-performance concrete. *Constr. Build. Mater.* **491**, 142682 (2025).

[52] He, Y., Chen, Q., Qi, C., Zhang, Q. & Xiao, C. Lithium slag and fly ash-based binder for cemented fine tailings backfill. *J. Environ. Manage.* **248**, 109282 (2019). 31374435 10.1016/j.jenvman.2019.109282

[53] Uysal, M., Yilmaz, K. & Ipek, M. The effect of mineral admixtures on mechanical properties, chloride ion permeability and impermeability of self-compacting concrete. *Constr. Build. Mater.* **27**, 263–270 (2012).

[54] Diamond, S. A review of alkali-silica reaction and expansion mechanisms 1. Alkalies in cements and in concrete pore solutions. *Cem. Concr. Res.* **5**, 329–345 (1975).

[55] Lacombe, C., Vidal, T., Sellier, A., Noret, C. & Anthiniac, P. Compressive creep of a concrete affected by advanced alkali-aggregate reaction. *Constr. Build. Mater.* **421**, 135627 (2024).

[56] Zhou, S., Zhang, Z. & Zhu, Y. Effect of lithium slag on hydration behavior of Portland cement paste. *Constr. Build. Mater.* **463**, 138909 (2025).

[57] He, Y. et al. Mechanical and environmental characteristics of cemented paste backfill containing lithium slag-blended binder. *Constr. Build. Mater.* **271**, 121567 (2021).

[58] Tan, H. et al. Preparation for micro-lithium slag via wet grinding and its application as accelerator in Portland cement. *J. Clean. Prod.* **250**, 119528 (2020).

[59] Alarcon-Ruiz, L., Platret, G., Massieu, E. & Ehrlacher, A. The use of thermal analysis in assessing the effect of temperature on a cement paste. *Cem. Concr. Res.* **35**, 609–613 (2005).

[60] Tan, H. et al. Utilization of lithium slag as an admixture in blended cements: Physico-mechanical and hydration characteristics. *J. Wuhan Univ. Technol. -Mat Sci. Edit*. **30**, 129–133 (2015).

[61] Barnett, S. J., Macphee, D. E., Lachowski, E. E. & Crammond, N. J. XRD, EDX and IR analysis of solid solutions between thaumasite and ettringite. *Cem. Concr. Res.* **32**, 719–730 (2002).

[62] Rahman, S. A. et al. Assessment of lithium slag as a supplementary cementitious material: Pozzolanic activity and microstructure development. *Cem. Concr. Compos.* **143**, 105262 (2023).

[63] Dong, P., Ahmad, M. R., Chen, B., Munir, M. J. & Saleem Kazmi, S. M. Preparation and study of magnesium ammonium phosphate cement from waste lithium slag. *J. Clean. Prod.* **316**, 128371 (2021).

[64] Scrivener, K. L., Juilland, P. & Monteiro, P. J. M. Advances in understanding hydration of Portland cement. *Cem. Concr. Res.* **78**, 38–56 (2015).

[65] Wang, J., Han, L., Liu, Z. & Wang, D. Setting controlling of lithium slag-based geopolymer by activator and sodium tetraborate as a retarder and its effects on mortar properties. *Cem. Concr. Compos.* **110**, 103598 (2020).

[66] Way, S. J. & Shayan, A. Early hydration of a portland cement in water and sodium hydroxide solutions: Composition of solutions and nature of solid phases. *Cem. Concr. Res.* **19**, 759–769 (1989).

